# A new open, high-resolution, multishell, diffusion-weighted imaging dataset of the living squirrel monkey

**DOI:** 10.1038/s41597-023-02126-x

**Published:** 2023-04-20

**Authors:** Thomas Orset, Julie Royo, Mathieu David Santin, Pierre Pouget, Michel Thiebaut de Schotten

**Affiliations:** 1 Brain Connectivity and Behaviour Laboratory, Sorbonne University, Paris, France; 2 Sorbonne University, Inserm U1127, CNRS UMR7225, UM75, ICM, Movement Investigation and Therapeutics Team, Paris, France; 3 ICM, Centre de NeuroImagerie de Recherche – CENIR, Paris, France; 4 Groupe d’Imagerie Neurofonctionnelle, Institut des Maladies Neurodégénératives-UMR 5293, CNRS, CEA University of Bordeaux, Bordeaux, France

**Keywords:** Phylogenetics, Brain, Cognitive neuroscience

## Abstract

Although very well adapted to brain study, Magnetic Resonance Imaging (MRI) remains limited by the facilities and capabilities required to acquire data, especially for non-human primates. Addressing the data gaps resulting from these limitations requires making data more accessible and open. In contempt of the regular use of *Saimiri sciureus* in neuroscience research, *in vivo* diffusion has yet to be openly available for this species. Here we built and made openly available a unique new resource consisting of a high-resolution, multishell diffusion-weighted dataset in the anesthetized *Saimiri sciureus*. The data were acquired on 11 individuals with an 11.7 T MRI scanner (isotropic resolution of 400 µm^3^). This paper presents an overview of our dataset and illustrates some of its possible use through example analyses. To assess the quality of our data, we analyzed long-range connections (whole-brain tractography), microstructure (Neurite Orientation Dispersion and Density Imaging), and axon diameter in the corpus callosum (ActiveAx). Constituting an essential new resource for primate evolution studies, all data are openly available.

## Background & Summary

Since the 1960s, the common squirrel monkey (*Saimiri sciureus*) has been used as a model in a wide range of biomedical fields, including cardiovascular physiology, pharmacology, genetics, and, to a great extent, neuroscience^1^. Squirrel monkeys are small non-human primates part of the Haplorhhini order and Cebidae family and, consequently, are closer to humans than Marmosets or Three shrews. They show phylogenic, anatomic, functional, and behavioral similarities with humans, making them very relevant to clinical neuroscience^2^.

Squirrel monkeys can be tested on various behavioral tasks adapted from rodents or non-human primates, including reaching, grasping, and inhibitory motor tasks^3^. They express hand preference in specific tasks^4^. Like macaques, they can achieve oculomotor tasks such as saccades or smooth pursuit tasks^5^. Working memory is another well-developed function in squirrel monkeys, comparable to macaques and humans^6^ and superior to marmosets^7^. Exploring the squirrel monkey’s brain can shed light on the mechanisms supporting primates’ functions and their evolutions as well as potentially reveal new targets for treating human brain dysfunctions^8,9^.

The squirrel monkey’s brain is more prominent (~22572 mm3) and has a higher neo-cortex/all brain ratio (69%) than the marmoset’s and the tree shrew’s respectively (~7241 mm3 and 60%) and (~3495 mm3 and ~27%)^10,11^. The squirrel monkey’s brain presents marked sulci, such as the arcuate sulcus. Finally, despite their larger brain than marmosets, squirrel monkeys’ global size remains suitable for small-bore MRI scanners working at ultra-high fields to peer into the anatomical and functional micro-structures of the living brain.

The relationship between anatomy and function is complex. From the microstructure to the large-scale network of task-related activations, investigating the brain circuitry is an elegant way to bridge anatomy with function^12^. Diffusion-weighted magnetic resonance imaging (DWI)^13^ is the only method sensitive to the orientation and fibers in the living brain. Combined with tractography, it reveals the close relationship or connection between brain regions^14^. DWI is a non-invasive Magnetic Resonance Imaging (MRI) method that can estimate the orientation of the diffusion of water molecules in any tissue. Because water molecules diffuse preferentially along and within axons rather than across them, tractography can produce plausible macroscopic white matter trajectories by piecing together local estimates of diffusion orientation^15–18^.

DWI can also be employed to estimate microstructural features such as tract-specific integrity^19^, neurite density^20^ and axonal diameter^21^, amongst others. Such connectivity metrics are optimal in the living brain as death quickly degrades the fragile white matter architecture^22^. Death affects mean diffusivity due to dehydration, protein cross-linking^23^ or decreased permeability^24^. Accordingly, diffusion in *ex vivo* models decreases and is less sensitive^25,26^. Temperature changes also alter diffusion properties in a compartment-dependent manner^24^. Further, long-term post-mortem brain conservation requires formalin fixation that is known to cause tissue shrinkage^27,28^, potentially in a non-homogenous way, as previously reported in the mouse^29^. Hence, the clinical relevance of DWI requires acquiring specimens *in vivo* for comparability to humans, in which proper perfusion and fixation are not feasible^30^. Since DWI sequences can be expensive to implement and challenging to process, several groups offered open access to datasets in humans^31^, macaques^32,33^, mice and other species^34–37^ to fuel progress in neurosciences. However, the absence of a living squirrel monkey DWI dataset hampers the progress of comparative models and prevents us from building comprehensive and accurate evolutionary trees of the brain.

Therefore, this paper summarizes the parameters and open access details to the first diffusion-weighted imaging dataset of the living squirrel monkey. We also provide codes, when available, preliminary results, and perspectives for future projects using this new resource.

## Methods

### Animals

Data were acquired from 11 adult female *Saimiri sciureus* between 6 and 10 years old at the imaging time, with an average weight of 754.5 ± 51 g (see Table 1 for individual details). All procedures were designed in association with the veterinarian team of the ICM Paris Brain Institute (agreement number B75-13-19), approved by the Regional Ethical Committee for Animal Experiments (Charles Darwin CE005 under the project reference APAFIS #21086-2019061415485300). Saimiris were housed in a social group within Paris Brain Institute (ICM) in a multi-compartment cage complex (17.5 m^3^) with structural and manipulable enrichments (ladders, platforms, hammock…) at constant temperature (24–26 °C), relative humidity (55%) and a 12 h light-darkness cycle. The animals had *ad libitum* access to water, and food was given three times a day, including commercial monkey chow, fruits, vegetables and other various enrichments (eggs, nuts, seeds, mealworms…).

**Table 1:** Age and weight details at the date of MRI for the 11 subjects available to date. Subjects were all adult females aged 6 to 10 years old and weighing 754.5 ± 51 g.

Animal Identification	Age (years)	Weight (g)
Agi	7	730
Asa	9	650
Cor	6	790
Ena	7	770
Gai	8	760
Gal	10	795
Iye	8	800
Kan	8	790
Maz	6	790
Ros	9	740
Wyn	7	675

### Anesthesia

Anesthesia was induced with an intramuscular (IM) injection of Alfaxalone (3 mg/kg) and maintained with isoflurane during animal preparation. The subject received an intramuscular injection of Dexdomitor (0.5 mg/kg) and anesthesia was maintained with perfusion of Alfaxalone (12 mg/kg). Internal temperature and respiratory rate were monitored throughout the MRI sequences. After 2 hours in the scanner or if the respiratory rate rose over 60/min, Alfaxalone’s flow rate was adjusted to 18 mg/kg to prevent awakening. A warm water flow maintained body temperature between 34.5 and 38.5 °C. From induction to awakening, anesthesia lasted 6 ± 0.5 hours.

### Acquisitions parameters

Magnetic Resonance (MR) Images were acquired using a Biospec USR 117/16 (Bruker, Germany). Radiofrequency emission and signal reception was performed using a 72-mm birdcage transceiver (Bruker, Germany). Before image acquisition, the automatic shim was computed using the MAPSHIM routine on an ellipsoid that fits the whole squirrel monkey brain. The field map obtained after shimming was estimated at 72*72*72 mm^3^ to perform later distortion correction. Diffusion-weighted images were then acquired using a Fat-Saturated 3D-DW segmented EPI sequence with the following parameters: 4 segments, TR/TE = 200/24 ms, Bandwidth = 600 kHz, Mtx = 160*160*128, FOV = 64.0*64.0*51.2 mm^3^ leading to an isotropic resolution of 400*400*400 µm^3^. Diffusion gradient duration δ = 3 ms and gradient separation Δ = 11 ms. A total number of 100 non-collinear diffusion gradient directions were acquired using three different b-values: 2 000, 1 000, and 300 s/mm2 with 64, 29, and 7 directions, respectively. Directions were generated offline on a sphere using Carruyer’s online Q-Space sampling tool^38^. A total number of 8 non-diffusion weighted volumes were also acquired. The whole scanning time for this multi-shelled diffusion acquisition was 3h04 min per animal.

### Preprocessing

**Fig. 1:**
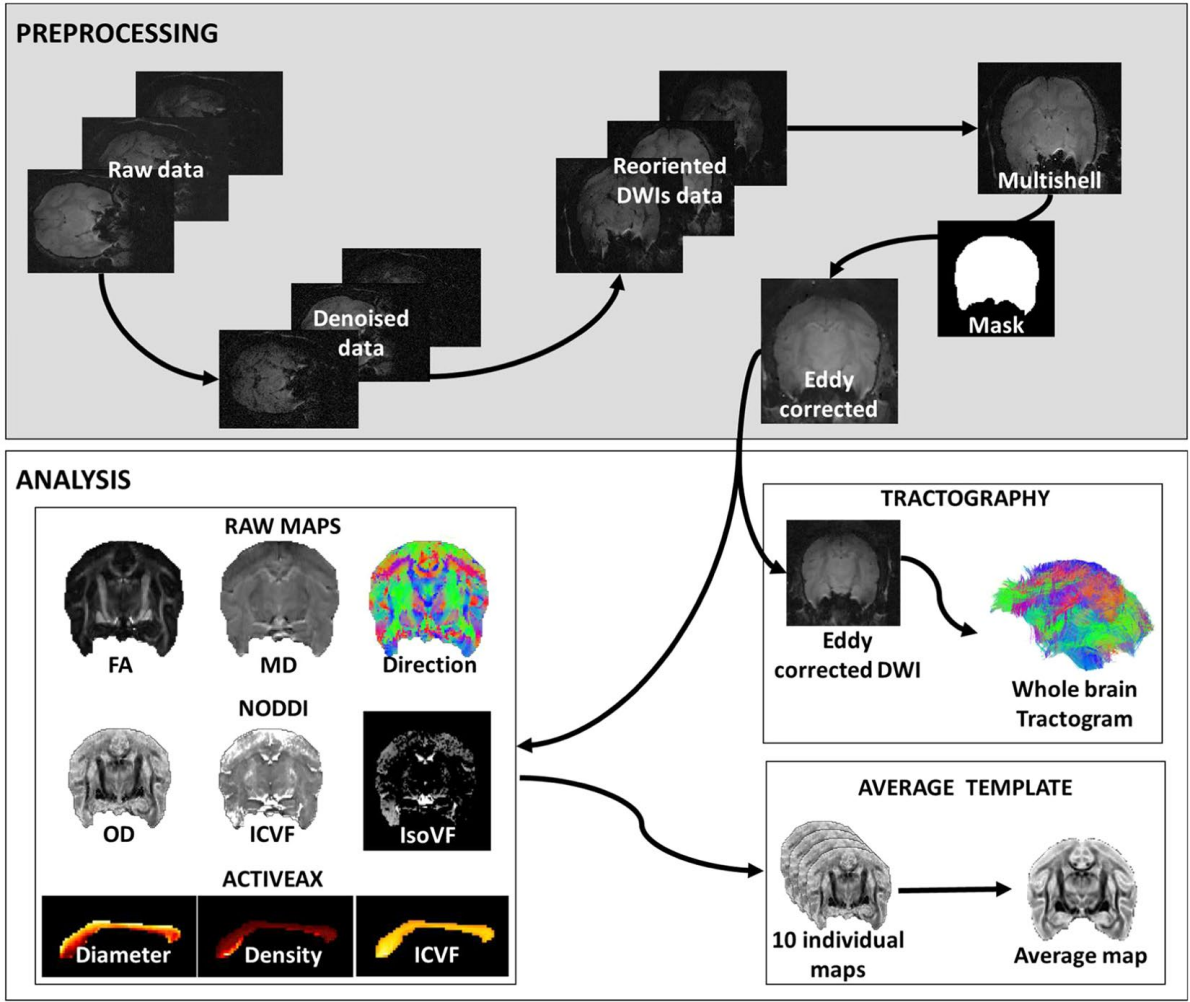
Overview of the experimental workflow. The top frame summarizes preprocessing steps from raw data to eddy-corrected multishell. The bottom frame outlines analyses applied to the preprocessed images. Eddy-corrected images are used to generate maps and compute tractography. Maps of 10 individuals were used to create average templates^46^. Note that the 11th individual data were acquired after the template creation and were not voluntarily excluded from the template creation.

Raw data were denoised, reoriented using ExploreDTI (www.exploredti.com), and the three b-values were merged (Fig. 1). A brain mask was generated and manually corrected with BrainSuite21a (www.brainsuite.org). Manual editing mainly consisted of extending the mask to the missing areas (due to a signal drop) or eroding the masked cerebrospinal fluid. Eddy currents-induced distortions and movement were corrected using the eddy tool from FSL.

### Tractography

StarTrack (http://www.mr-startrack.com), together with Matlab, performed deterministic tractography analysis on multishell diffusion-weighted images by means of the damped Richardson Lucy Spherical deconvolution algorithm^39,40^ (Fig. 1). Spherical deconvolution parameters were set to ALFA = 2, iterations = 1000, n = 0.001, and r = 8. Whole brain deterministic tractography was performed using two runs of the M-Euler algorithm, with an absolute threshold of 0.005, step size of 0.4 mm, angle threshold of 35° and min and max streamline length of 8 and 100 mm, respectively. Manual tractography dissections were performed on the monkey Gal using Trackvis (www.trackvis.org) to demonstrate feasibility.

### Models fitting

DWI can shed light on advanced microstructural information through specific models.

For instance, neurite orientation dispersion and density imaging (NODDI) estimates the complexity of dendrites and axons based on diffusion MRI data^20^ (Fig. 1). It relies on three sub-model for intracellular, extra-cellular and cerebrospinal fluid (CSF) compartments. NODDI analyses provide maps of isometric volume fraction in CSF, intracellular volume fraction, and orientation dispersion (OD). The latter is a surrogate for neurite dispersion and synaptic complexity. Additionally, ActiveAx is a model used to calculate an orientationally invariant index of axon diameter and density^41^. Accordingly, we applied both models to the multishell dataset (B300, B1000, and B2000) after eddy current correction. Note that for practical purposes, ActiveAx estimations focused on the corpus callosum’s mid-section (delineated manually by the authors).

## Data Records

The present dataset is stored on Figshare^42^ and a dedicated website, which will be regularly updated with additional data and analyses (http://saimiri.bcblab.com). We also opened a forum topic for questions and answers (https://neurostars.org/t/squirrel-monkey-brain-mapping/24110). The dataset comprise 11 folders, one per subject. Each subject folder contains one subfolder per b-value (2 000, 1 000, 300) and one subfolder for the B0 map registered to the diffusion data set. B-values folders comprise the raw image in .nii format, the b-vecs file in .txt format, and metadata in a .json file. B0 map folder includes B0 maps in .nii format and metadata in a .json file.

## Technical Validation

We present here the first DWI open database of living squirrel monkeys’ brains. To prompt the quality of our data we assessed the details of small anatomical structures, the quality of tractography dissections, the clarity of advanced multishell diffusion maps and the reproduction of classical expected brain patterns.

**Fig. 2:**
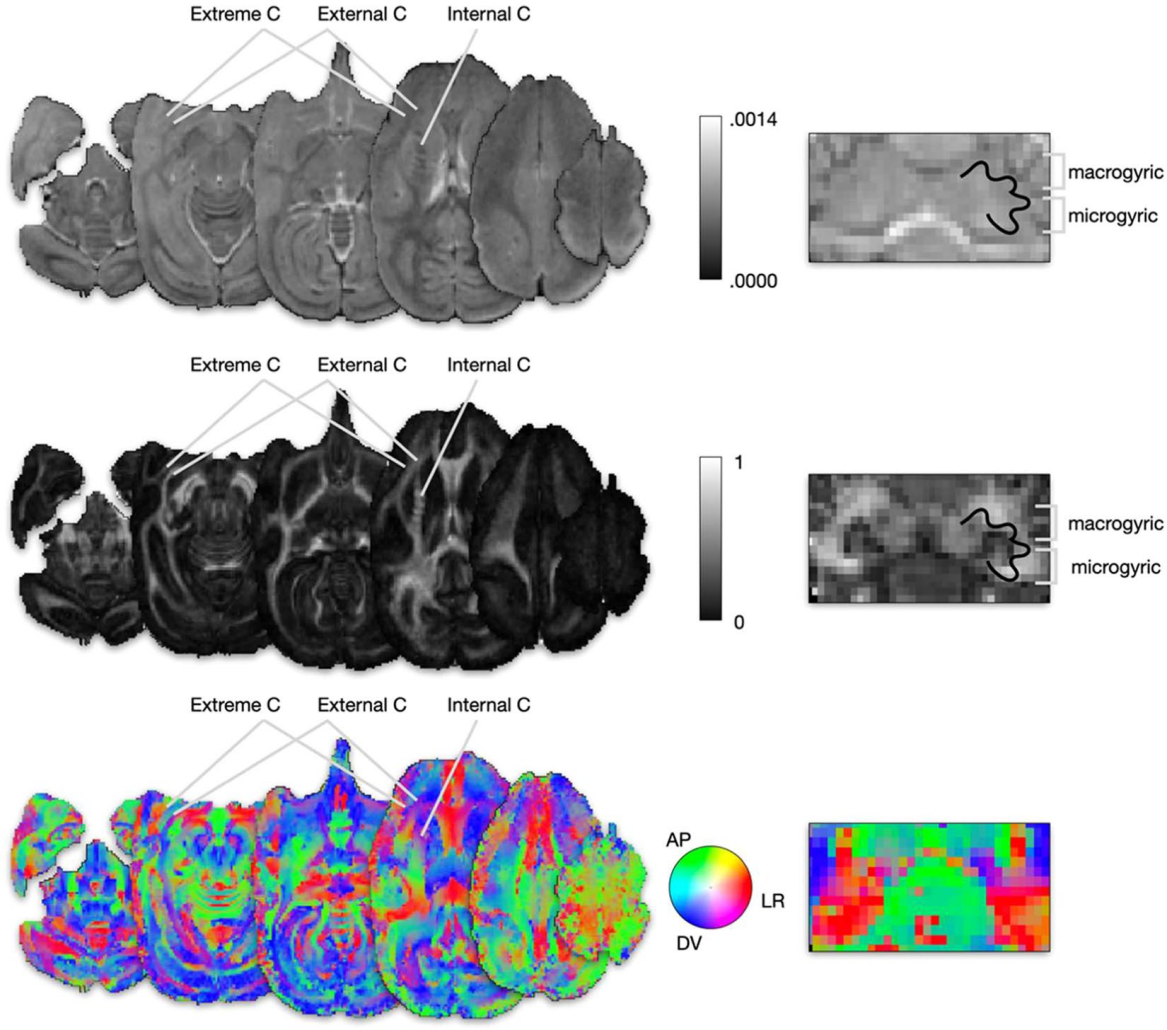
Raw DWI maps N = 1. Axial (left panel) and coronal cerebellar (right panel) sections of (**a**) Mean Diffusivity, (**b**) Fractional Anisotropy and (**c**) RGB-encoded principal diffusion direction.

Figure 2 shows that despite the high resolution of the dataset, the signal was strong enough to make anatomical structures visible at the cortical and subcortical levels. Fractional anisotropy maps sharply delineated fine white matter anatomical landmarks such as the distinction between the extreme and external capsule, stripes in the internal capsule and the micro- and macrogyric architecture of the dentate nucleus^43^.

**Fig. 3:**
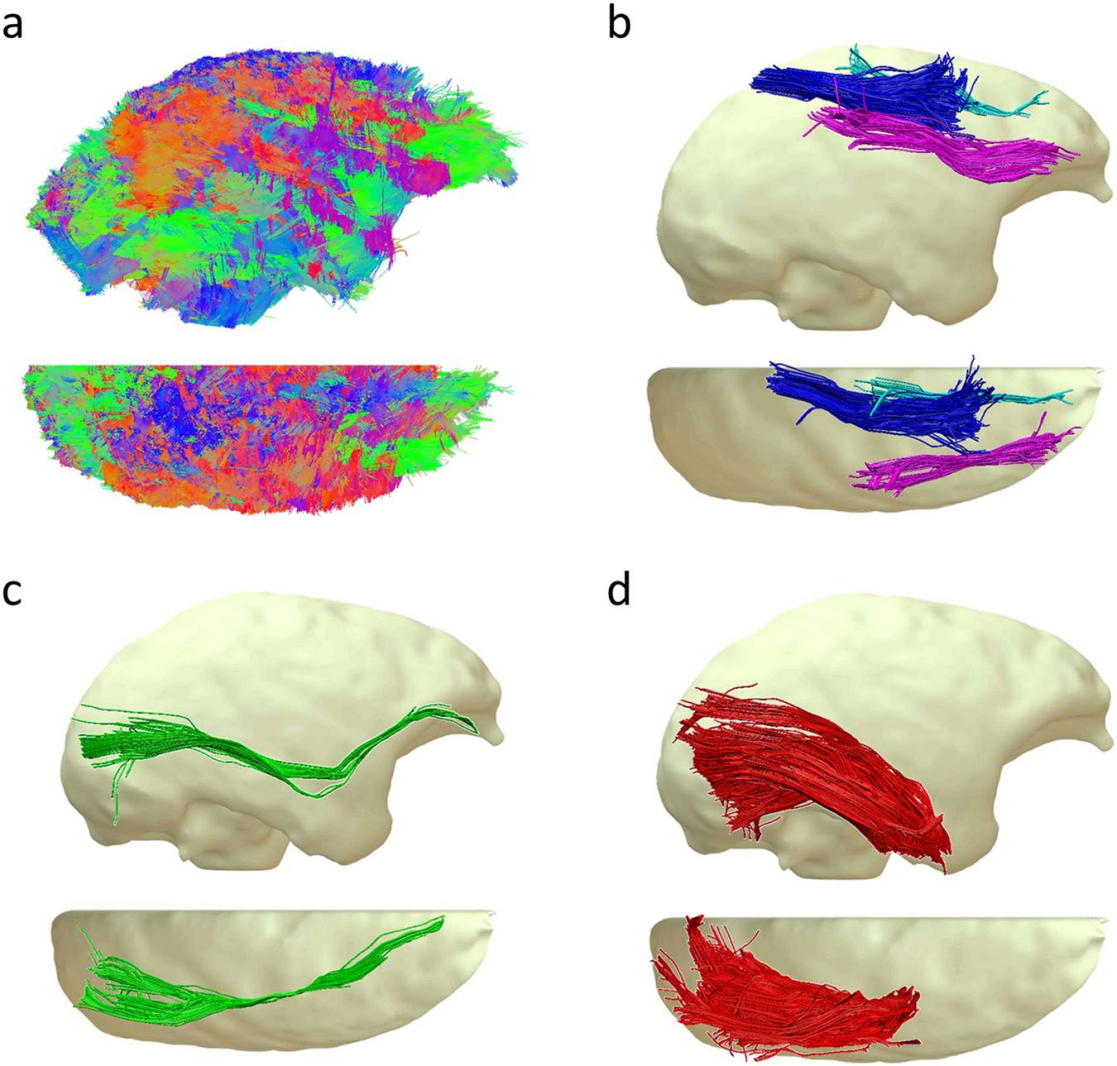
Tractography dissection N = 1. (**a**) Right and top view of a whole brain tractogram composed of 554360 tracks. (**b**) The three branches of the Superior longitudinal fasciculus (SLF) I, II and III . (**c**) Inferior Longitudinal Fasciculus (IFOF) . (**d**) Inferior Longitudinal Fasciculus (ILF) .

Figure 3 shows the result of deterministic whole-brain tractography and examples of track dissection in a single subject. The whole tractogram (Fig. 3a) was composed of 554360 streamlines and allowed us to dissect thin fibers bundles such as the three SLF branches (Fig. 3b) as well as long-range connections, namely the inferior fronto-occipital fasciculus (Fig. 3c). The dissection of the inferior longitudinal fasciculus (Fig. 3d) has revealed a dense bundle with scattering in its posterior part.

The tractography of the squirrel monkey brain revealed a set of projection, association, and commissural connections quite comparable to humans. However, trained anatomists could spot obvious differences, including an indisputable disproportion between the ventral (inferior longitudinal fasciculus and inferior fronto-occipital fasciculus) and the dorsal (fronto-parietal system) when compared to humans^44,45^. These differences reflect squirrel monkeys’ relatively smaller frontal and parietal lobes than humans^46–48^. Despite these disparities, the complexity of the squirrel monkey’s circuitry was persuasive enough to investigate limbic, fronto-parietal, perisylvian and visual systems with potential translation to humans at the macroscopic level.

**Fig. 4:**
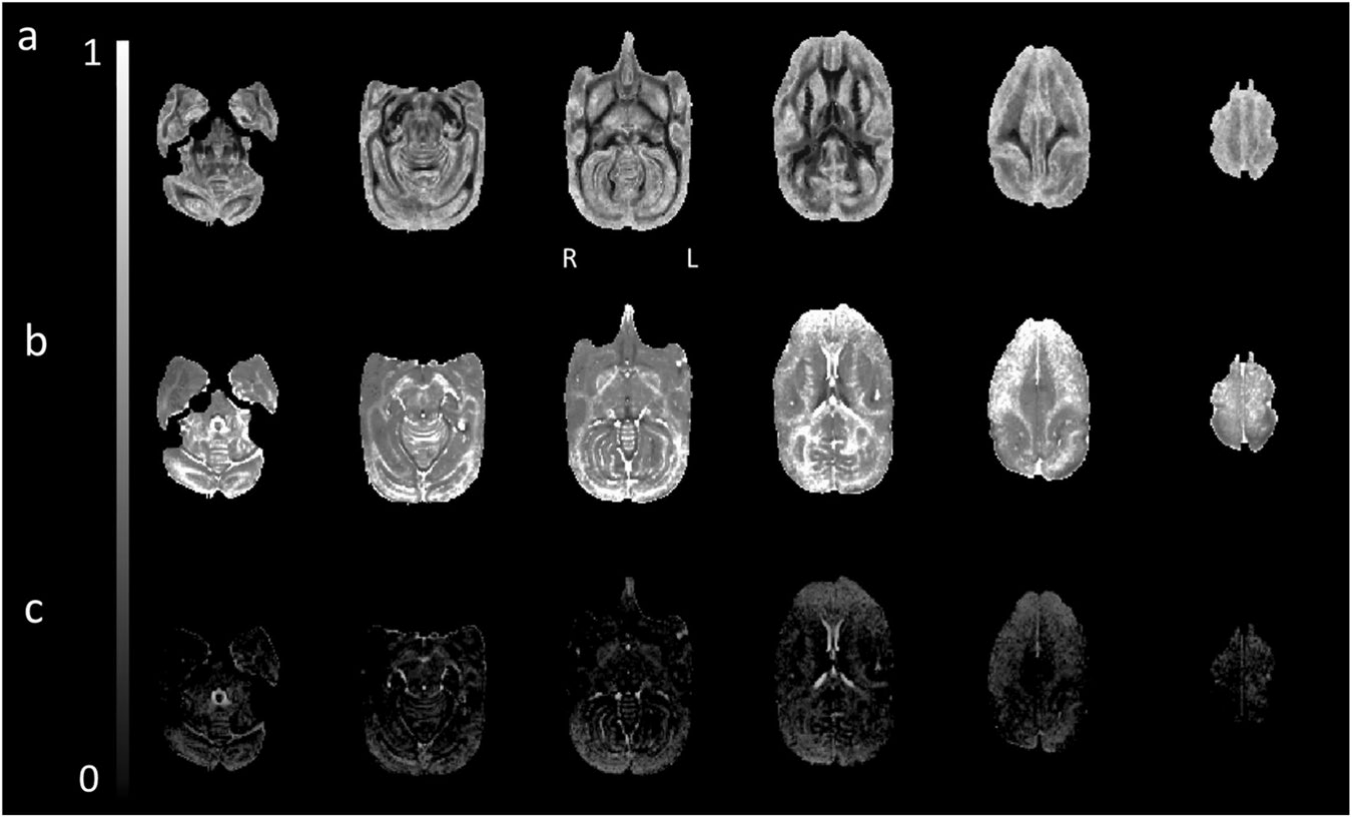
NODDI analysis N = 1. Axial view from a whole brain NODDI analysis for (**a**) Orientation Dispersion, (**b**) Intracellular Volume Fraction, and (**c**) Isotropic (CSF) Volume Fraction.

Our multishell acquisition allows NODDI to estimate cerebrospinal volume fraction, neurite density, and dispersion at the microscopic level. Figure 4 displays the maps computed for a single subject. As expected, the Orientation Dispersion map (Fig. 4a) highlighted grey matter structures consistent with their complex axonal and dendritic (i.e., neurite) diffusion profile. Reversely, the map for intracellular volume fraction (Fig. 4b) displays consistent patterns with higher values in white matter. The third map for isotropic volume fraction (Fig. 4c) showed maximum values in the ventricles.

**Fig. 5:**
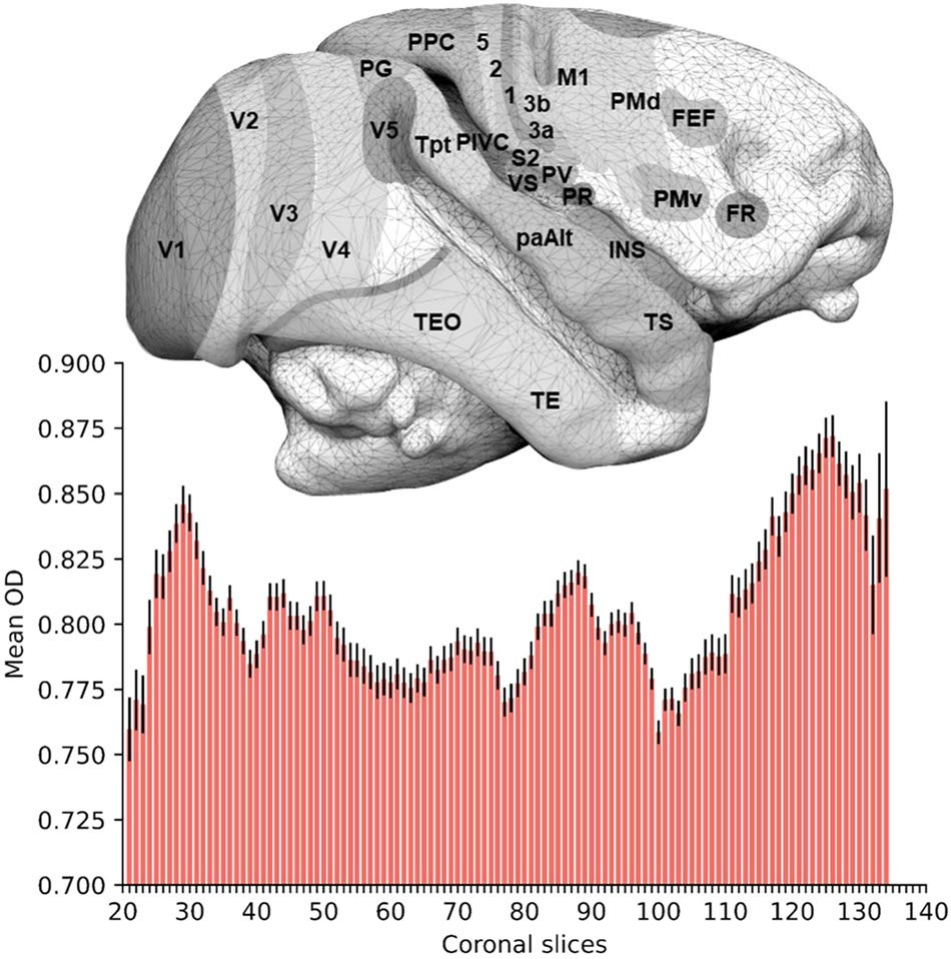
Mean Orientation Dispersion by slices N = 10. A right lateral view of a Saimiri brain surface (top, modified from Royo *et al*. 2021) is aligned to a histogram (bottom) representing the average orientation dispersion (OD) value for grey matter in each coronal slice. Error bars represent the standard error. FEF frontal eye field, FR frontal rostral field, INS insula, M1 primary motor area, PaAlt lateral parakoniocortex, PIVC parieto-insular vestibular cortex, PG inferior parietal lobule, PMv premotor area ventral, PMd premotor area dorsal, PPC posterior parietal cortex, PR presumptive parietal rostral, PV parietal ventral, S2 secondary somatosensory area, 1 2 3a 3b and 5 cytoarchitectonic areas 1 2 3a 3b and 5, TE temporal region, TEO temporo-occipital region, Tpt temporo-parietal region, TS temporalis superior cortex, V1 V2 V3 V4 and V5 visual areas 1 2 3 4 and 5, VS ventral somatosensory.

In order to assess the potential for our measurements to investigate variations in the cortical architecture of the squirrel monkey, we computed a mean OD template from 10 individuals using Advanced Normalisation Tools (ANT) together with the function *buildtemplateparallel.sh*. The mean OD value for the grey matter for each coronal slice was extracted and plotted in Fig. 5. It reveals a clear gradient of variations on the anterior-posterior axis, notably in the occipital and frontal areas. Neurite orientation dispersion being an indicator of the local connective properties of the cortex, this variation indicates the existence of an anterior-posterior gradient of complexity in the frontal area. It might align with brain evolutionary theories of progressive expansion^49,50^ and cytoarchitectural^51,52^ and neuroimaging^53,54^ gradients in the frontal lobes. A similar gradient, posterior-anterior this time, seems to exist in the occipital lobes, albeit, to our knowledge, less documented.

**Fig. 6:**
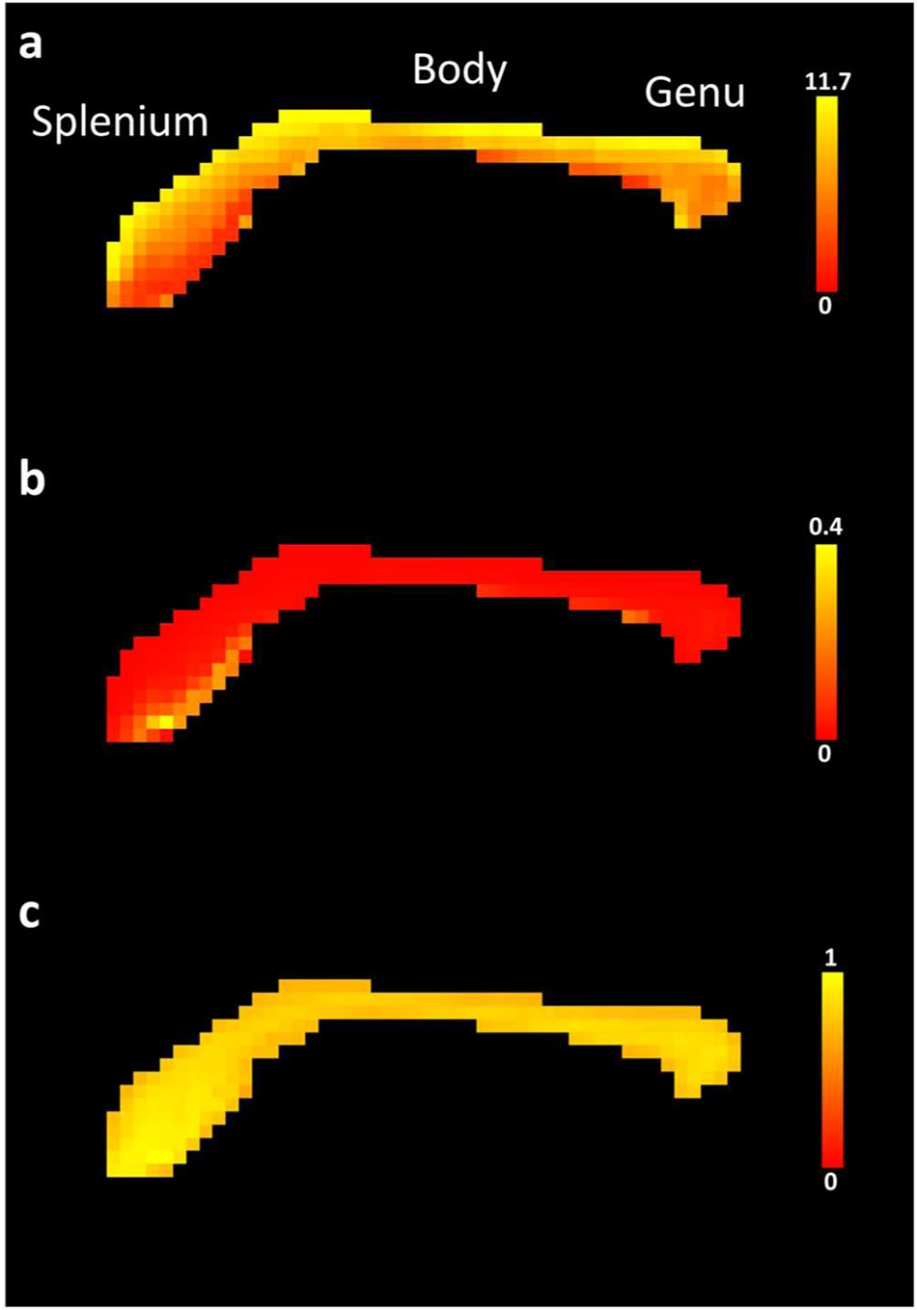
ActiveAx analysis N = 1. Close-up of sagittal mid-sections of corpus callosum maps for (**a**) mean axonal diameter in µm, (**b**) axonal density in axons/µm^2^ and (**c**) Intra-cellular volume fraction. Mean axonal diameter, axonal density and ICVF correspond to ActiveAx generated files FIT_a, FIT_d and FIT_v respectively.

The multishell parameter of our acquisition also allows for using ActiveAx to estimate mean axonal density and diameter. Figure 6 is an example of ActiveAx analysis focusing on a sagittal mid-section of the corpus callosum and demonstrating a dorsoventral gradient of axonal diameter with thicker axons located dorsally. Importantly, DWI-derived estimation of the axonal diameter profile received a lot of criticism^55^. Accordingly the values estimated by AxtiveAx were higher than standard values reported post-mortem in other species^56^. While postmortem shrinking can affect values, future investigation on our dataset will allow us to provide true postmortem estimation of the axonal diameter in the squirrel monkey. These post-mortem investigations in the same individuals will allow for the validation of the biological inferences derived from neuroimaging methods.

Despite reaching a staggering resolution of 400 µm *in vivo*, estimation of the orientation and microstructure of connections derived from diffusion-weighted imaging is still not exempt from limitations. In particular, it is essential to highlight that the resolution of myelinated axons (1 to 6 µm) leads to partial volume effects that hamper a perfect estimation when a bundle of connections crosses, fans or merges. In spite of this limitation, we provided an estimate of the connections orientation, neurite density and axonal diameter derived from some of the best algorithms currently available for people to explore and potentially improve analyses shortly.

## Usage Notes

Our *in vivo* diffusion-weighted imaging dataset of living squirrel monkeys is available on Figshare and a dedicated website regularly updated with additional data and analyses (http://saimiri.bcblab.com). We also opened a forum topic for questions and answers (https://neurostars.org/t/squirrel-monkey-brain-mapping/24110). This dataset provides future perspectives for anatomists working on brain evolution, who will be able to peer further into the past of primate brain evolution. It will benefit neuroscientists who wish to investigate behavioral models or anatomical systems not available otherwise and neuroimagers who would like to test their new algorithms. This resource will also permit the refinement of evolutionary mechanisms modeling and potentially allow for identifying future therapeutic targets. We believe this dataset, combined with other resources available to researchers (e.g., PRIME-DE), will permit sharper phylogenetical investigations.

## Data Availability

All custom codes used to analyze the data are shared on GitHub: https://github.com/Orset-Thomas/Squirrel_monkey_DWI.git.
